# THBS1/CD47 Modulates the Interaction of γ-Catenin With E-Cadherin and Participates in Epithelial–Mesenchymal Transformation in Lipid Nephrotoxicity

**DOI:** 10.3389/fcell.2020.601521

**Published:** 2021-02-18

**Authors:** Li Gao, Ting-ting Yang, Jun-sheng Zhang, Hong-xia Liu, Dong-cheng Cai, Lin-tao Wang, Jing Wang, Xin-wei Li, Kun Gao, Su-ya Zhang, Yu-jia Cao, Xiao-xia Ji, Miao-miao Yang, Biao Han, Sheng Wang, Lu He, Xiao-yan Nie, Dan-mei Liu, Gang Meng, Chao-yong He

**Affiliations:** ^1^State Key Laboratory of Natural Medicines, Department of Pharmacology, China Pharmaceutical University, Nanjing, China; ^2^Pathophysiology Department, The Fourth Affiliated Hospital of Anhui Medical University, Hefei, China; ^3^Anhui Yizhiben Center for Judicial Expertise, Hefei, China; ^4^Center for Scientific Research of Anhui Medical University, Hefei, China

**Keywords:** cd47, E-cadherin, γ-catenin, fibrosis, inflammation, lipid nephrotoxicity

## Abstract

Hyperlipidemia, an important risk factor for cardiovascular and end-stage renal diseases, often aggravates renal injury and compromises kidney function. Here, histological analysis of human kidney samples revealed that high lipid levels induced the development of renal fibrosis. To elucidate the mechanism underlying lipid nephrotoxicity, we used two types of mouse models (Apoe^−/−^ and C57BL/6 mice fed a 45 and 60% high-fat diet, respectively). Histological analysis of kidney tissues revealed high-lipid-induced renal fibrosis and inflammation; this was confirmed by examining fibrotic and inflammatory marker expression using Western blotting and real-time polymerase chain reaction. Oxidized low-density lipoprotein (OX-LDL) significantly induced the fibrotic response in HK-2 tubular epithelial cells. RNA-sequencing and Gene Ontology analysis of differentially expressed mRNAs in OX-LDL-treated HK-2 tubular epithelial cells and real-time PCR validation in Apoe^−/−^ mice showed that the expression of thrombospondin-1 (*THBS1*) in the high-fat group was significantly higher than that of the other top known genes, along with significant overexpression of its receptor CD47. *THBS1* knockdown cells verified its relation to OX-LDL-induced fibrosis and inflammation. Liquid chromatography tandem mass spectrometry and STRING functional protein association network analyses predicted that THBS1/CD47 modulated the interaction between γ-catenin and E-cadherin and was involved in epithelial–mesenchymal transition, which was supported by immunoprecipitation and immunohistochemistry. CD47 downregulation following transfection with small-hairpin RNA in OX-LDL-treated tubular epithelial cells and treatment with anti-CD47 antibody restored the expression of E-cadherin and attenuated renal injury, fibrosis, and inflammatory response in OX-LDL-treated cells and in type 2 diabetes mellitus. These findings indicate that CD47 may serve as a potential therapeutic target in long-term lipid-induced kidney injury.

## Introduction

Hyperlipidemia has emerged as a major health concern worldwide. In China alone, the prevalence of hyperlipidemia was as high as 41.9% in 2014 (Huang et al., 2014). Karr et al. reported a cholesterol imbalance in more than 100 million individuals (~53% adults) in the United States. Evidence suggests that excessive lipid intake contributes to conditions, such as fatty liver, coronary atherosclerosis, and lipid nephrotoxicity. In the United States, fewer than 50% of the individuals with elevated low-density lipoprotein (LDL-C) levels receive treatment, and among those receiving treatment, <35% achieve control (Karr, 2017). Therefore, further research is warranted to understand the pathogenesis of hyperlipidemia.

The “Lipid Nephrotoxicity Hypothesis” was established in 1982. It suggested that hyperlipidemia, resulting from compensatory hepatic synthesis of lipoproteins in response to urinary loss of albumin, contributes to the progression of glomerulosclerosis and tubulo-interstitial fibrosis. Successive studies have investigated nephrotoxicity. Dyslipidemia is not only a consequence of chronic kidney disease (CKD) but also a cause of renal damage. Experiments have confirmed that cholesterol supplementation in the diet can result in the development of various glomerular diseases (Du and Ruan, 2019). Kuwahara et al. recently suggested the involvement of lipotoxic compounds filtered by the glomeruli in injuries associated with high-fat diet (HFD)-induced kidney disease (Vergès, 2015; Kuwahara et al., 2016). There are two sources of lipotoxic compounds. First, abnormal innate renal or renal immune cells, which can induce the development of lipid metabolism disorders and ectopic lipid accumulation (ELA), e.g., phospholipid accumulation in enlarged lysosomes and impaired autophagic flux in kidney epithelial cells in obese patients (Yamamoto et al., 2017). Chen et al. have suggested that the expression of an enzyme catalyzing the conversion of citrate to acetyl CoA was induced in overweight or obese patients with CKD and was responsible for increasing ELA and promoting CKD progression (Chen et al., 2019). Adipose differentiation-related protein and sterol regulatory element binding protein-1 (SREBP-1) have been reported to be upregulated in the kidney during diabetic kidney disease and are thought to mediate lipid accumulation and tubular damage in the kidneys of individuals with diabetes (Guebre-Egziabher et al., 2013). Second, lipids present in circulation, i.e., serum free fatty acids (FAs) and oxidized low-density lipoproteins (OX-LDLs) (Du and Ruan, 2019). In diabetic nephropathy, FAs bound to plasma albumin are filtered through the glomeruli into the urine, thus inducing the development of tubulointerstitial injury (Tanaka et al., 2016). Other studies have also indicated that excessive lipids in circulation undergo oxidative modification, resulting in the binding of oxidized lipids to glycosaminoglycans in the glomerular basement membrane, thereby increasing its permeability and promoting the development of tubulointerstitial disease (Guebre-Egziabher et al., 2013). These processes are detrimental to renal function. Accumulated lipids may be involved in the development of nephrotoxicity, i.e., increased permeability of the glomeruli and initiation or aggravation of tubulointerstitial fibrosis (TIF) (Moorhead et al., 1982). Several mechanisms, such as epithelial–mesenchymal transition (EMT) observed in CKD, are known to underlie TIF (Tanaka et al., 2016; Gao et al., 2020). The epithelium is characterized by the presence of tight junctions, which maintain its integrity and stability. Cells undergoing EMT lose the expression of key epithelial markers such as E-cadherin and acquire mesenchymal markers, such as fibronectin, collagen I (Col-1), and α-smooth muscle actin (α-SMA) (Gao et al., 2018; Seccia et al., 2019). A critical step during EMT is the “cadherin switch,” which involves a decrease in the expression of E-cadherin, a protein that is essential for maintaining the epithelial cell phenotype (Gumbiner, 2005; Mezi et al., 2017). Other proteins involved in the “cadherin switch” are α-, β-, and γ-catenins that bind to the cytoplasmic domain of E-cadherin and link the catenin/cadherin complex to intracellular cytoskeleton actin fibers. β-catenin activates the Wnt signaling pathway following dissociation from E-cadherin in the cytoplasm and translocates into the nucleus as a cofactor. Thus, it is associated with cell survival, proliferation, metastasis, and EMT (Reya and Clevers, 2005; Wang W. et al., 2019). In addition, γ-catenin, known as junction plakoglobin (JUP), interacts with the cytoplasmic domain of cadherins (McEwen et al., 2014). In colorectal cancer cells, the C457 modification of γ-catenin results in impaired migration and proliferation of cells by affecting the “cadherin switch” (Conacci-Sorrell et al., 2002; Kim et al., 2017). However, it is unclear whether the catenin/cadherin complex influences the progression of EMT in lipid nephrotoxicity and therefore warrants confirmation.

CD47 is a member of the immunoglobulin superfamily that is expressed by several cell types. This transmembrane receptor mediates self-recognition (releasing a “don't eat me” signal) and prevents clearance by phagocytic cells, which recognize CD47 via the counter-receptor signal regulatory protein alpha (SIRPα) (Kojima et al., 2016; Alvey and Discher, 2017). In addition, CD47 and its proteoglycan isoform serves as a receptor for binding to the matricellular protein thrombospondin-1 (THBS1) (Kaur et al., 2011). Studies have shown that blockade of THBS1/CD47 signaling reduces oxidative injury, decreases inflammatory response (Wang et al., 2018), restores autophagy (El-Rashid et al., 2019), and renews epithelial cells in several models of acute kidney injury (Navarathna et al., 2015; Rogers et al., 2016; Xu et al., 2018). Furthermore, CD47^−/−^ mice treated with a CD47 blocking antibody have been reported to exhibit amelioration of fibrotic histological changes. Furthermore, plasma THBS1 levels are associated with CKD (Julovi et al., 2020). Therefore, in this study, we investigated the role of THBS1/CD47 signaling in lipid nephrotoxicity and determined whether CD47 can serve as an effective therapeutic target.

## Materials and Methods

### Model of Lipid Nephrotoxicity

#### Human Samples

All evaluated human tissues were obtained from The Fourth Affiliated Hospital of Anhui Medical University and Anhui Yizhiben Center for Judicial Expertise in China in 2020. In this study, tissues from six patients with hyperlipidemia were used. Three of these patients had coronary atherosclerosis and fatty liver disease. The other three patients had dyslipidemia; for these patients, tissue samples were derived from distant portions of renal cell carcinomas and served as controls. Tissue sections were fixed with 4% paraformaldehyde (PFA). The Ethics Committee of the Fourth Affiliated Hospital of Anhui Medical University approved the study.

#### Mouse Models

All animal experiments were performed at the China Pharmaceutical University in Jiangsu Province, China, and the experimental procedures were approved by the China Pharmaceutical University Ethical Committee on Animal Experiments.

Apoe^−/−^ mice with a C57BL/6 background were obtained from the Jackson Laboratory and housed at a constant temperature (22.5 ± 0.5°C; 50 ± 5% humidity) under a 12-h dark/light cycle. Male Apoe^−/−^ mice (8 weeks old) were fed a normal diet (ND; 12.8% kilocalories: fat, 5%; protein, 23%; carbohydrate, 55%) or HFD (fat, 35%; carbohydrate, 45%; protein, 20%) (Trophic Animal Feed High-tech Co. Ltd., Nanjing, China) for 12 weeks and subsequently euthanized under anesthesia. We also established a second model of lipid nephrotoxicity using male C57BL/6 mice (8 weeks old) fed ND (12.8% kilocalories: fat, 5%; protein, 23%; carbohydrate, 55%) or HFD (fat, 60%; carbohydrate, 20%; protein, 20%) for 12 weeks. Another model of lipid nephrotoxicity, i.e., type 2 diabetes mellitus (DM), was established. Male C57BL/6 mice (6 weeks old) were fed ND (12.8% kilocalories: fat, 5%; protein, 23%; carbohydrate, 55%) or HFD (fat, 60%; carbohydrate, 20%; protein, 20%) for 6 weeks. HFD and control groups were administered a high dose of streptozocin (STZ; 100 mg/kg, intraperitoneal injection, once) and vehicle (citrate buffer), respectively. The mice were considered diabetic when the blood glucose level exceeded 250 mg/dl. After 4 weeks, the diabetic and control mice were divided into four groups (*n* = 6–8 per group) in the following manner: IgG-treated control mice (CT-IgG), anti-CD47 antibody-treated control mice (CT-CD47-Ab), IgG-treated diabetic mice (DM-IgG), and anti-CD47 antibody-treated diabetic mice (DM-CD47-Ab). Subsequently, 200 μg of anti-CD47 antibody (Invitrogen, USA) or IgG (Invitrogen, USA) was administered to diabetic or control mice, respectively, by tail vein injection once every 2 days for another 4 weeks (Kojima et al., 2016). Mice were anesthetized with sodium pentobarbital (50 mg/kg intraperitoneally), and kidney tissue and blood samples were collected for further experiments.

### Reagents and Materials

Antibodies against CD47 and γ-catenin were obtained from Abcam (Cambridge, UK), while those against Col-1, α-SMA, THBS1, and β-actin were procured from Santa Cruz Biotechnology (Dallas, TX, USA). Rabbit anti-E-cadherin was purchased from Bioss Biotechnology (Beijing, China) and antibodies against vimentin and CD68 were supplied by MXB Biotechnologies (Fuzhou, China). Lipofectamine 2000 was purchased from Science Biotechnology (Invitrogen, Beijing, China) and the Protein Assay Kit was purchased from Beyotime Institute of Biotechnology (Jiangsu, China). Masson's trichrome (Masson) and Van Gieson (VG) staining kits were procured from Zhuhai Besso Biotechnology Institute (Wuhan, China). Leucine-serine-lysine-leucine (LSKL), a competitive TGF-β1 antagonist and an inhibitor of thrombospondin, was procured from MedChemExpress (MCE, Shanghai, China). Kits for the triglyceride (TG) assay, total cholesterol (TC) assay, LDL cholesterol assay, high-density lipoprotein cholesterol assay, and blood urea nitrogen (BUN) assay were purchased from Nanjing Jiancheng Bioengineering Institute.

### Cell Culture

The human kidney tubular epithelial cell line HK-2 was cultured in 5% fetal bovine serum (FBS)-supplemented Gibco Dulbecco's modified Eagle's medium (DMEM)/F12 at 37°C in a humidified 5% CO_2_ atmosphere. After 12 h of starvation using DMEM/F12 medium containing 0.5% FBS, HK-2 cells were treated with 25 μg/ml OX-LDL (Yiyuan Biotechnology, Guangzhou, China) for 48 h (Sastre et al., 2013). Following treatment with 10 μg/ml anti-CD47 antibody or anti-IgG antibody or a combination of these antibodies with 50 μM LSKL for 12 h (Willet et al., 2013), HK-2 cells were stimulated with OX-LDL. The treated cells were harvested for further analyses, including Western blotting, real-time PCR, and immunofluorescence (IF). Three or four *in vitro* experiments were independently performed.

### Western Blotting

Tissues or cells were lysed in ice-cold radioimmunoprecipitation assay buffer. The bicinchoninic acid assay (BCA) protein kit (Yesen, Shanghai, China) was used to quantify protein concentration. After loading samples on 10% sodium dodecyl sulfate polyacrylamide gel electrophoresis (SDS-PAGE) gels, the resolved proteins were transferred onto nitrocellulose membranes (Millipore, Massachusetts, USA). The membranes were probed for 12 h at 4°C using antibodies against Col-1, α-SMA, CD47, E-cadherin, and β-actin, and then probed with secondary antibodies (Zsbio, Beijing, China) for 1.5 h at 37°C. After washing with Tween, the blots were developed using a chemiluminescence method (Thermo Scientific, Waltham, MA, USA). The results were quantified using ImageJ 1.45s software (NIH, Bethesda, MD, USA).

### RNA Extraction and Real-Time PCR

Total RNA was extracted from kidney homogenates or HK-2 cells using TRIzol reagent (Takara, Kusatsu, Japan) in accordance with the manufacturer's instructions. RNA concentration was evaluated using a NanoDrop 2000 Spectrophotometer (Thermo Scientific). RNA, nuclease-free water, and RealMasterMix (Yesen, Shanghai, China) were used for cDNA synthesis. RT-PCR was performed using the Hieff UNICON® qPCR SYBR Mix (Yesen, Shanghai, China). The detection system was used as previously described (Gao et al., 2018; Liu et al., 2020). The following primer sequences were used:

Human fibronectin, forward 5′-TACCAAGGTCAATCCACACCCC-3′
reverse 5′-CAGATGGCAAAAGAAAGCAGAGG-3′
Human α-SMA, forward 5′-ATCAAGGAGAAACTGTGTTATGTAG-3′
reverse 5′-GATGAAGGATGGCTGGAACAGGGTC-3′
Human Col-l, forward 5′-TCTAGACATGTTCAGCTTTGTGGAC-3′
reverse 5′-TCTGTACGCAGGTGATTGGTG-3′
Human CD47, forward 5′-AGAAGGTGAAACGATCATCGAGC-3′
reverse 5′-CTCATCCATACCACCGGATCT-3′
Human β-actin, forward 5′-CGCCGCCAGCTCACCATG-3′
reverse 5′-CACGATGGAGGGGAAGACGG-3′
Mouse fibronectin, forward 5′-CCGCCGAATGTAGGACAAGA-3′
reverse 5′-GCCAACAGGATGACATGAAATG-3′
Mouse α-SMA, forward 5′-CGGGCTTTGCTGGTGATG-3′
reverse 5′-CCCTCGATGGATGGGAAA-3′
Mouse Col-l, forward 5′-TGTAAACTCCCTCCACCCCA-3′
reverse 5′-TCGTCTGTTTCCAGGGTTGG-3′
Mouse β-actin, forward 5′-CATTGCTGACAGGATGCAGAA-3′
reverse 5′-ATGGTGCTAGGAGCCAGAGC-3′
Real-time PCR data were analyzed using the 2^−ΔΔCt^ method.

### RNA-Sequencing

RNA-seq was conducted as previously described (Wang D. et al., 2019). Total RNA was isolated using TRIzol reagent according to the manufacturer's protocol. Total RNA with RNA integrity number > 7.0 was processed following evaluation using the RNA 6000 Nano LabChip Kit (Agilent, California, USA). Poly (A) RNA was purified from 1 μg of total RNA using Dynabeads Oligo (Salajegheh et al.) 25–61005 (Thermo Fisher, CA, USA) by two rounds of purification and then fragmented under the following conditions: 94°C for 5–7 min. The cleaved RNA fragments were reverse transcribed to create a final cDNA library according to the manufacturer's protocol (mRNA Sequencing Sample Preparation Kit, Illumina). The average insert size for the cDNA library was 300 ± 50 bp. We performed 2 × 150 bp end sequencing (PE150) on an Illumina Novaseq 6000 (LC-Bio Technology Co., Ltd., Hangzhou, China) according to the manufacturer's instructions. Differential expression analysis was performed based on adjusted *p*-values; volcano plots were prepared to depict fold-change differences in gene expression. Gene Ontology (GO) enrichment and Kyoto Encyclopedia for Genes and Genome (KEGG) analyses of the differentially expressed mRNAs were also conducted. RNA-sequencing data were submitted to Gene Expression Omnibus (GEO) [https://www.ncbi.nlm.nih.gov/geo/query/acc.cgi?acc=GSE161737].

### Immunoprecipitation

HK-2 cells were harvested and washed thrice with ice-cold PBS as previously described (Wang et al., 2020). The cells were lysed with IP buffer (Beyotime Biotechnology, Shanghai, China) supplemented with a complete protease inhibitor cocktail (Yesen, Shanghai, China) on ice for 30 min. Cell lysates were collected and prepared for IP. Protein G Sepharose beads (MedChemExpress, Shanghai, China) were incubated with 10 μl of anti-CD47 (1 mg/ml) or anti-γ-catenin (1 mg/ml) for 4 h at 4°C with constant shaking. After washing, the beads were incubated with the prepared proteins at 4°C for 12 h. Immunoprecipitated proteins were subjected to gel electrophoresis or liquid chromatography tandem mass spectrometry (LC-MS/MS).

### LC-MS/MS

Immunoprecipitated proteins were excised from SDS-PAGE gels and digested as previously described (Chou et al., 2018). Briefly, the excised gel pieces were washed twice in 50% acetonitrile (ACN) for 30 min and 100% ACN for 30 min. Following reduction and recovery, the samples were digested by enzymes for 12 h at 37°C. The supernatant was collected and extracted. Dried peptides were dissolved in high-performance liquid chromatography (HPLC) buffer for desalination. The samples were then subjected to LC-MS/MS analysis using a Thermo High Performance Liquid Chromatograph (Easy-nLC1200, Thermo Scientific) and a high-resolution mass spectrometer (Q Exactive plus, Thermo Scientific). The data were submitted to a database that searched against the sequence library in the Uniport-reviewed *Homo sapiens* (Human) database using Proteome Discover 2.4.

### Immunofluorescence

The harvested cells were fixed with 4% PFA for 5 min, washed with PBS, and incubated with 10% donkey serum for 60 min at 37°C. Next, the cells were incubated overnight at 4°C with Col-1/α-SMA antibody (1:200) and treated with fluorescein isothiocyanate-labeled secondary antibody (Bioss, Beijing, China). Nuclei were stained with 4,6-diamidino-2-phenylindole (DAPI). After a final washing step with PBS, the sections were imaged using a fluorescence microscope (Leica, Germany).

### Lentivirus Package and Transfection

The plasmids, Control, THBS1-SH1 (CCGAAAGGGACGATGACTATG), THBS1-SH2 (CTATGCTATCACAACGGAGTT), THBS1-SH3 (TGACATCAGTGAGACCGATTT), psPAX2, and pMD2.G were extracted using the E.Z.N.A. Endo-free Plasmid Maxi Kit, and their concentrations were measured using the NanoDrop 2000. HEK-293T cells were seeded at a density of 5 × 10^6^ cells/ml in a six-well culture plate (NUNC, Thermo scientific, USA) and incubated at 37°C with 5% CO_2_. After overnight incubation, the medium was changed to serum-free DMEM, and then the transfection was performed with the following cocktail for each transfection in sterilized 1.5-ml tubes: 2 μg OF pLKOG/pLKOshRNA plasmid, 1.4 μg of psPAX2 packaging plasmid, and 0.6 μg of pMD2.G envelope plasmid to 200 μl of serum-free DMEM medium. Next, 5 μl of Turbofect transfection reagent (Thermo Scientific, USA) was added to each tube. After incubating for 20 min, the mixture was added dropwise to each well and incubated at 37°C in a 5% CO_2_ incubator. After 4–6 h, the medium was gently changed to DMEM + 10% FBS without antibiotics medium. After overnight culture, 0.5 ml of DMEM + 10% FBS medium without antibiotics was added to each well. After 24 h, the medium was harvested from the cells and filtered through a 0.45-μm filter to remove the cells.

HK-2 cells were seeded at a density of 1.0 × 10^5^ cells/ml in 48-well plates for lentivirus infection. The original medium containing the lentivirus was added to the HK-2 cells. After 6 h, the medium was replaced with growth medium. The cells were cultured for 48 h. Then, the cells were cultured with medium containing 10 μg/ml puromycin (sc-108071, Santa, USA) for 2 days. Finally, the positive cells were collected for further study.

### Short-Hairpin RNA Interference

CD47 was silenced by transfecting cells with specific shRNA or negative control shRNA (GeneChem, Shanghai, China) using Lipofectamine 2000 according to the manufacturer's instructions. Cells were screened for CD47 knockdown using puromycin and cultured in DMEM/F12 supplemented with 5% FBS. shRNA transfection efficiency was determined by checking CD47 expression using Western blotting and real-time PCR.

### Kidney Histology

Human and mouse kidney tissues were fixed (4% PFA), dehydrated, and embedded in paraffin. Tissue sections (5 μm thick) were stained with hematoxylin and eosin (H&E) to determine the degree of tissue damage and subjected to Masson and VG staining to evaluate the degree of fibrosis in accordance with the manufacturer's instructions (Zhuhai Besso Biotechnology Institute, Wuhan, China).

Immunohistochemistry (IHC) was performed for paraffin-embedded sections using a microwave-based antigen retrieval technique. IHC sections were incubated with rabbit anti-Col-1, anti-α-SMA, anti-E-cadherin, anti-CD47, and rabbit anti-CD68 antibody for 24 h at 4°C and then with secondary antibodies for 30 min at 37°C, followed by labeling with liquid chromogen 3,3-diaminobenzidine tetrahydrochloride (DAB). Then, sections were subjected to automatic digital slide scanning (KFBIO, Yuyao, China).

### Oil Red O Staining

Frozen tissue sections/HK-2 cells were fixed with 4% PFA for 15 min and washed thrice with PBS. After treatment with 60% isopropanol for 1–3 s, the sections were stained with Oil red O (Sigma, St. Louis, MO, USA) for 8–10 min. The sections were washed twice with 60% isopropanol and then thrice with PBS. The sections were subjected to automatic digital slide scanning (KFBIO).

### Statistical Analysis

Data are expressed as the mean ± standard error of mean (SEM). Significance was analyzed using one-way analysis of variance (ANOVA), followed by Tukey's *post-hoc* test using GraphPad Prism 5.0 software (GraphPad, La Jolla, CA, USA).

## Results

### Renal Fibrosis Was Observed in Tissues From Patients With Hyperlipidemia

**Figure 1:**
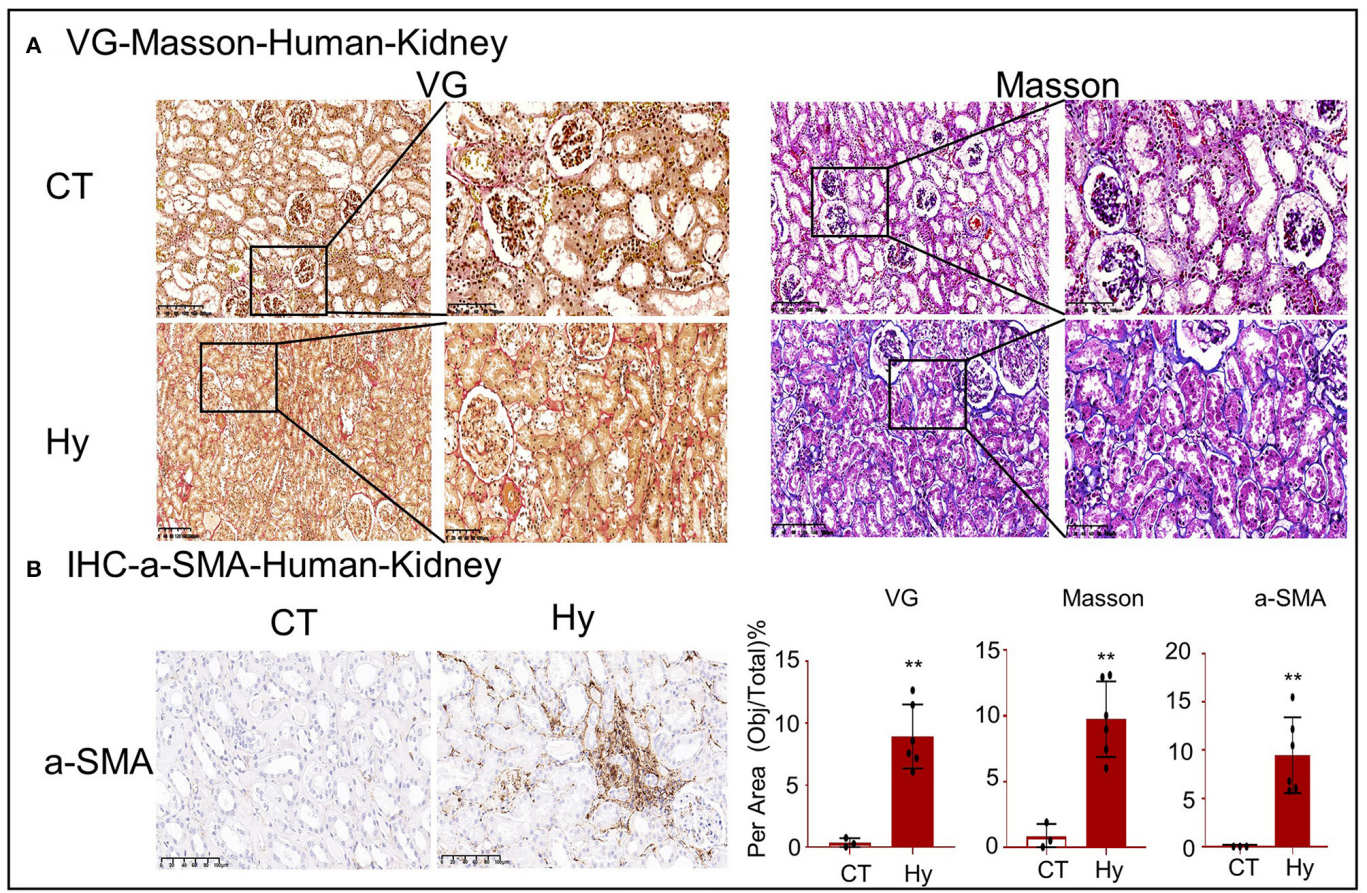
High-lipid-induced fibrotic response in human kidneys. **(A)** Van Gieson and Masson's trichrome staining of kidney sections. **(B)** Immunohistochemistry for α-SMA revealed high-lipid-induced renal fibrosis. CT, control (*n* = 3). Hy, human hyperlipidemia (*n* = 6). **p < 0.01 compared with the control.

To assess lipid nephrotoxicity, human renal tissues were obtained from six patients with hyperlipidemia and control patients (Table 1; Supplementary Figure 1A). VG and Masson staining and IHC for α-SMA revealed significant fibrosis in the kidney tissue following exposure to high concentrations of lipids (Figures 1A,B).

**Table 1:** Characteristics of Human Samples.

	**204268**	**203208**	**200799**	**192917**	**201149**	**201284**	**FQ036**	**FR014**	**FR018**
Age (yrs)	30	53	55	50	53	47	64	59	56
Gender	Male	Male	Male	Female	Female	Male	Female	Female	Male
Blood Pressure (mmHg)	138/89	109/72	108/69	139/88	141/84	111/69	122/72	122/80	-
Blood Glucose (mmol/L)	5.32	4.89	4.92	4.66	6.04	5.07	6.9	-	-
AST (u/L)	27	13	18	12	15	15	16	-	-
ALT (u/L)	30	11	16	8	17	9	21	-	-
BUN (mmol/L)	5.40	4.67	3.56	4.28	5.22	3.87	3.88	-	-
Cr (umol/L)	59.7	63.5	63.9	72.8	49.9	74.1	42	-	-
Coronary artery stenosis	-	-	-	-	-	-	IV	IV	IV
HDL (mmol/L)	1.05	1.54	1.74	0.86	1.52	0.97		-	
LDL (mmol/L)	3.19	1.89	1.74	2.20	4.85	2.21		-	
TC (mmol/L)	4.87	3.81	4.50	3.86	6.49	3.72		3.56	
TG (mmol/L)	1.56	0.82	0.85	2.23	2.05	1.94		2.77	
Fat Liver	-	-	-	+	+	+	30% Fat	40% Fat	50% Fat

*NO. 204268, NO. 203208, and NO. 200799 is control, NO. 192917, NO. 201149, NO. 201284, FQ036, FR014, and FR018 is hyperlipidemia group*.

### Establishment of Mouse Models With High-Lipid-Induced Renal Fibrosis

**Figure 2:**
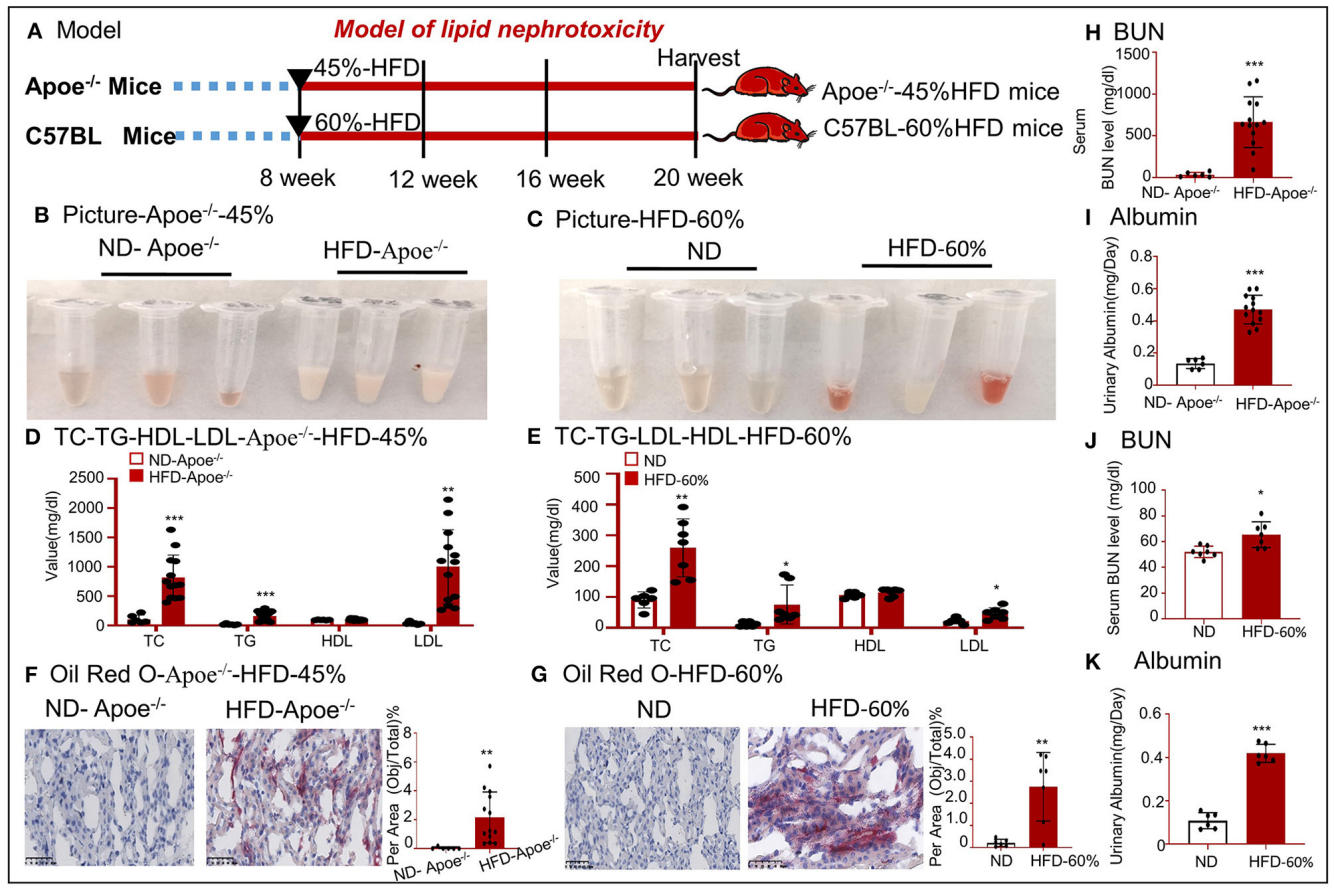
Establishment of two mouse models fed a high-fat diet. **(A)** Work flow for generation of two mouse models that were fed with a high-fat diet. **(B,C)** Image of the serum samples from HFD-fed mice. **(D,E)** Values of TC, TG, HDL, and LDL. **(F,G)** Oil red O staining of tissues from two mouse models. **(H,J)** Values of serum BUN. **(I,K)** Values of urinary albumin. *p < 0.05, **p < 0.01, ***p < 0.001 as compared with the control. ND-Apoe^−/−^, Apoe^−/−^ mice fed a normal diet. HFD-Apoe^−/−^-45%, Apoe^−/−^ mice fed a 45% high-fat diet. ND, C57BL/6 mice fed a normal diet. HFD-60%, C57BL/6 mice fed a 60% high-fat diet. Data are shown as mean ± SEM for 6–12 mice.

To further confirm lipid-mediated nephrotoxicity, renal tissues were obtained from Apoe^−/−^ mice fed a 45% HFD and C57BL/6 mice fed a 60% HFD. The workflow for the establishment of the two models is shown in Figure 2A. We found that the color of the serum changed and that the serum TC, TG, and LDL levels were significantly higher in mice with high-lipid-induced fibrosis than those in the control mice (Figures 2B–E). Oil red O staining demonstrated excessive lipid deposition in the kidneys of both mouse models (Figures 2F,G). BUN and urinary albumin levels were significantly higher in the model group than those in the control group (Figures 2H–K).

### High Lipid Levels Induced a Fibrotic Response in Mice and HK-2 Cells

**Figure 3:**
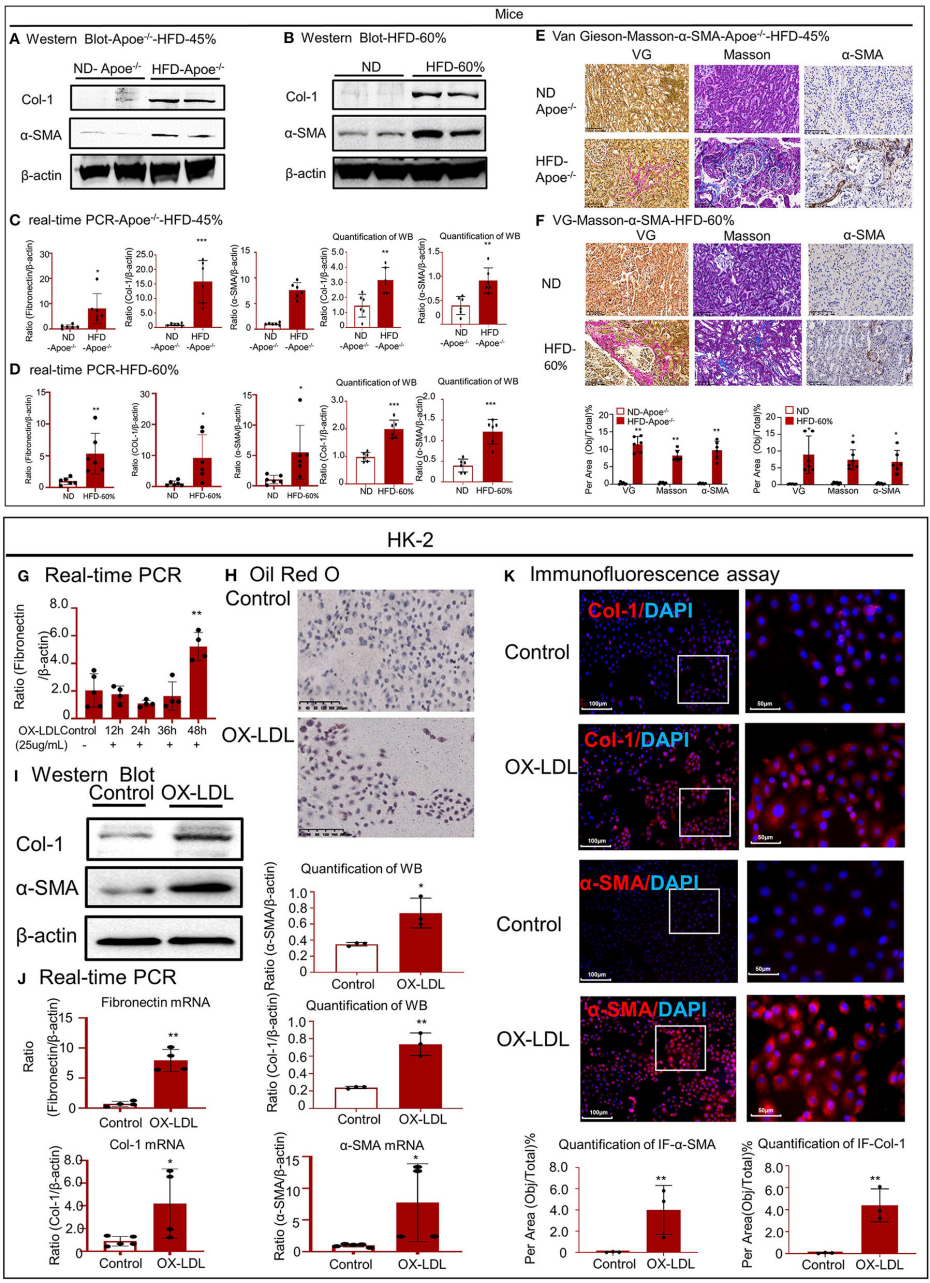
High-lipid-induced fibrotic response in mice and HK-2 cells. **(A,B)** Western blot analysis and quantitative data of Col-1 and α-SMA expression in two types of mouse models. **(C,D)** Real-time PCR of fibronectin, Col-1, and α-SMA in mice. **(E,F)** Van Gieson staining and Masson's trichrome staining of the kidney tissue revealed the presence of high-lipid-induced renal fibrosis, which was further confirmed by immunohistochemistry for α-SMA *in vivo*. **(G)** Cellular fibrosis model established using HK-2 cells treated with OX-LDL. **(H)** Oil red O staining of HK-2 cells following treatment with OX-LDL. **(I,J)** Western blotting and real-time PCR for checking the expression of fibrogenic genes. **(K)** Immunofluorescence to check the expression of Col-1 and α-SMA *in vitro*. *p < 0.05, **p < 0.01, ***p < 0.001 as compared with the control. ND-Apoe^−/−^, Apoe^−/−^ mice fed a normal diet. HFD-Apoe^−/−^-45%, Apoe^−/−^ mice fed a 45% high-fat diet. ND, C57BL/6 mice fed a normal diet. HFD-60%, C57BL/6 mice fed a 60% high-fat diet. Data are shown as mean ± SEM for six mice or three to four independent cell experiments.

Western blotting revealed that the expression of Col-1 and α-SMA increased in the high-lipid models (Figures 3A,B). Real-time PCR was conducted to detect the expression of fibronectin, Col-1, and α-SMA mRNA (Figures 3C,D). Accumulated lipids promoted kidney fibrosis, as confirmed by VG and Masson staining and IHC for α-SMA in the two mouse models (Figures 3E,F). We also established a cellular fibrosis model by treating HK-2 cells with 25 μg/ml OX-LDL for 48 h (Figure 3G). Oil red O staining revealed lipid deposition in HK-2 cells following treatment with OX-LDL (Figure 3H). Western blotting and real-time PCR revealed that the expression of fibrogenic genes (fibronectin, Col-1, and α-SMA) significantly increased following OX-LDL stimulation (Figures 3I,J). These observations were further supported by IF (Figure 3K).

### High Lipid Levels Induced Inflammatory Response in Mice and HK-2 Cells

**Figure 4:**
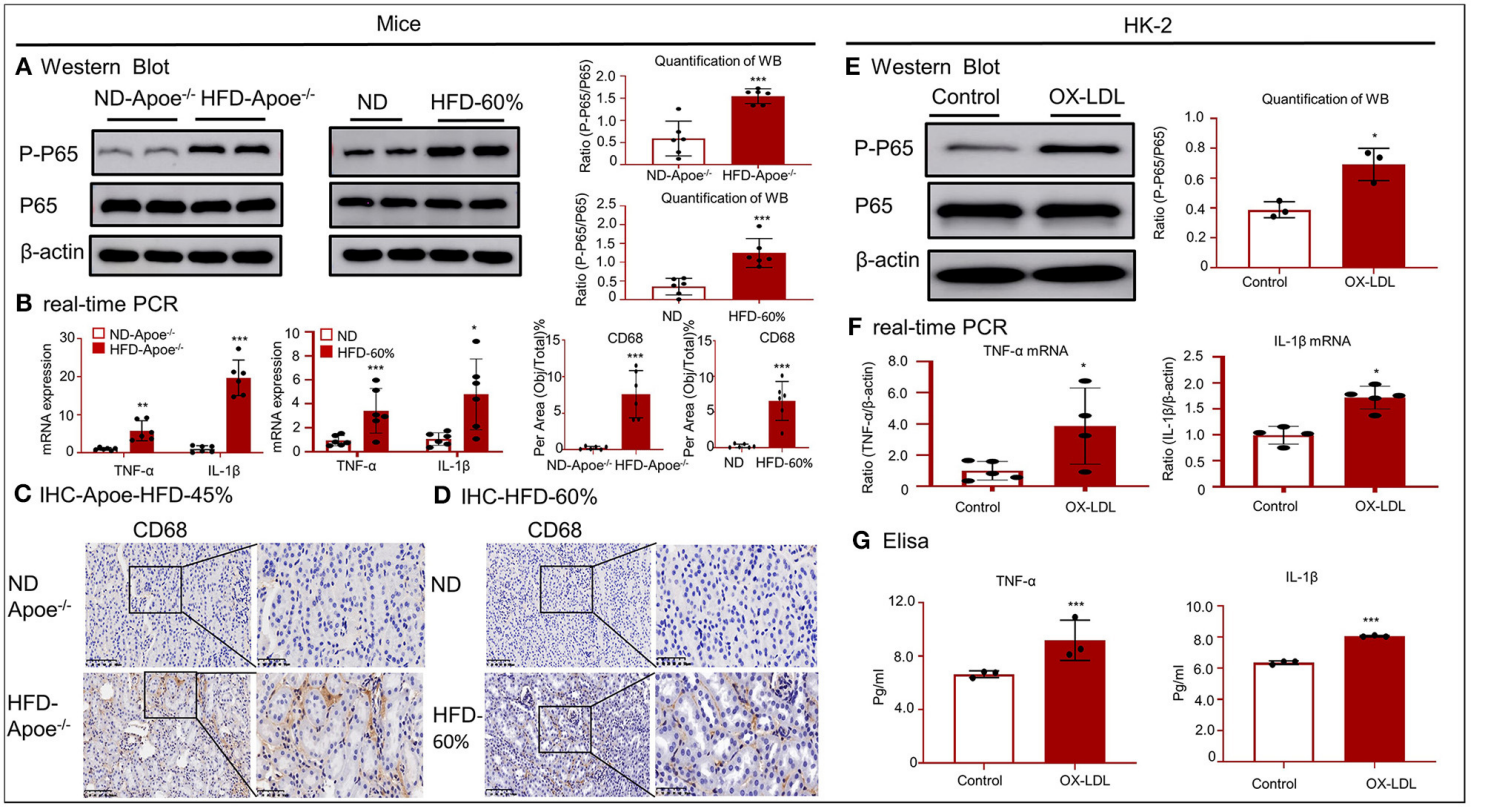
High-lipid-induced inflammation in mice and HK-2 cells. **(A,E)** Western blotting and quantification of p-P65 expression *in vivo* and *in vitro*. High lipid levels activated NF-κB-induced renal inflammation. **(B,F)** Real-time PCR for checking the expression of TNF-α and IL-1β in mice and HK-2 cells. **(C,D)** Immunohistochemistry for CD68 in two mouse models revealed the presence of a high lipid-induced inflammatory response. **(G)** ELISA for inflammatory cytokines, including TNF-α and IL-1 β. *p < 0.05, **p < 0.01, ***p < 0.001 compared with the control group. ND-Apoe^−/−^, Apoe^−/−^ mice fed a normal diet. HFD-Apoe^−/−^-45%, Apoe^−/−^ mice fed a 45% high-fat diet. ND, C57BL/6 mice fed a normal diet. HFD-60%, C57BL/6 mice fed a 60% high-fat diet. Data represent mean ± SEM for six mice or three to four independent cell experiments.

Western blotting and real-time PCR revealed that the accumulated lipids activated the nuclear factor kappa B (NF-κB) signaling pathway and increased the mRNA levels of tumor necrosis factor (TNF)-α and interleukin (IL)-1β (Figures 4A,B). As shown in Figures 4C,D, IHC for CD68 revealed the infiltration of many macrophages into the renal tissues of the two mouse models. These results indicate that accumulated lipids induced an inflammatory response in the two mouse models. The inflammatory response was aggravated in HK-2 cells after OX-LDL stimulation, consistent with the increase in the phosphorylation of p65 and expression of inflammatory cytokines, including TNF-α and IL-1β (Figures 4E–G).

### THBS1/CD47 Signaling Induced EMT in HK-2 Cells and Mice Fed HFD

**Figure 5:**
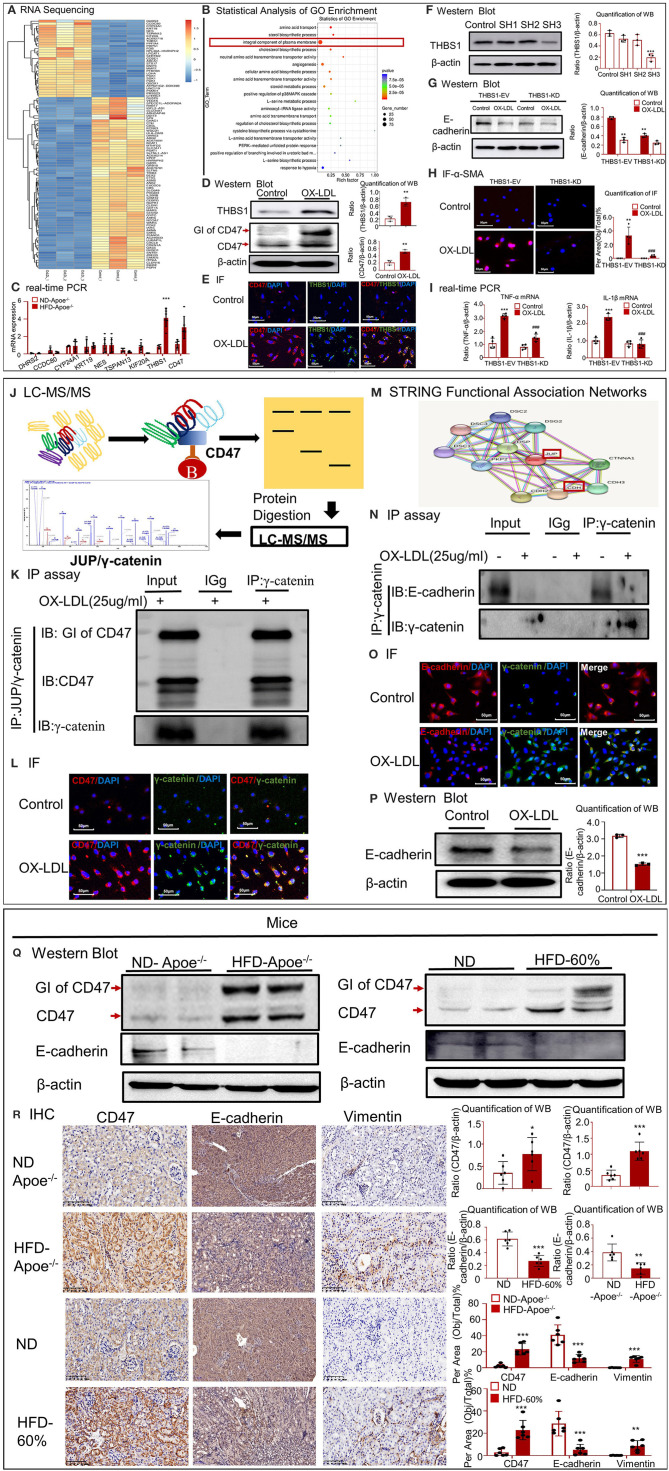
CD47 promoted EMT in high-lipid-treated HK-2 cells and mice. **(A)** RNA-seq of high-lipid-treated HK-2 cells. **(B)** Statistical analysis of GO enrichment. **(C)** Real-time PCR to check the expression of *DHRS2, CCDC80, CYP24A1, KRT19, NES, TSPAN13, KIF20A*, and *THBS1* and CD47 in Apoe^−/−^ mice. **(D)** Western blotting and quantification of THBS1, CD47, and GI of CD47. **(E)** IF for THBS1 and CD47. **(F)** Verification of THBS1 knockdown in HK-2 cells. The results demonstrated that THBS1 expression was downregulated after transfection of HK-2 cells with the packaged virus. **(G)** Western blotting and quantification of E-cadherin in THBS1 knockdown cells. **(H)** IF for α-SMA expression in THBS1 knockdown cells. **(I)** Real-time PCR to check the expression of TNF-α and IL-1β in THBS1 knockdown cells. **(J)** Mass fragmentation of amino acids following digestion of proteins immunoprecipitated (IP) using CD47 antibody. **(K)** IP assay results showed the interaction between CD47 and γ-catenin. **(L)** IF for CD47 and γ-catenin *in vitro*. **(M)** STRING functional association networks of γ-catenin. **(N)** IP assay further confirmed the interaction of E-cadherin with γ-catenin. **(O)** IF for E-cadherin and γ-catenin *in vitro*. **(P)** Western blotting and quantification of E-cadherin *in vitro*. **(Q)** Western blotting and quantification of CD47 and E-cadherin *in vivo*. **(R)** Immunohistochemistry for CD47, E-cadherin, and vimentin *in vivo*. *p < 0.05, **p < 0.01, ***p < 0.001 as compared with the control group. ###*p* < 0.001 compared with the THBS1-EV-OX-LDL group. GI of CD47, proteoglycan isoform of CD47. ND-Apoe^−/−^, Apoe^−/−^ mice fed a normal diet. HFD-Apoe^−/−^-45%, Apoe^−/−^ mice fed a 45% high-fat diet. ND, C57BL/6 mice fed a normal diet. HFD-60%, C57BL/6 mice fed a 60% high-fat diet. JUP, junction plakoglobin, also known as γ-catenin. CDH1, E-cadherin. EV, empty vector; KD, knockdown. Data are shown as mean ± SEM for six mice or three to four independent cell experiments.

To determine the mechanisms underlying high-lipid-induced fibrosis and inflammation, RNA-seq was performed in HK-2 cells under OX-LDL stimulation (Figures 5A,B). In total, 1054 genes were identified as differentially expressed genes (DEGs) with a fold change > 2 or fold < 0.5 and *p*-value < 0.05 using R package edgeR or DESeq2. Among the DEGs, 477 were upregulated and 577 were downregulated. RNA-seq confirmed that the expression of many inflammatory genes (including *IL1R1, CXCL16*, and *CSF1R*) substantially increased after OX-LDL treatment. Meanwhile, functional clustering was conducted using GO analysis; the results revealed that the genes associated with the integral components of the plasma membrane displayed a unique expression profile. Several DEGs mapped to the plasma membrane were enriched for the biological function term, cell–cell adhesion (*CLDN15, CLDN16, CLDN1, CDH4, PCDH17, PCDH1*, and *PCDHGA7*), and *TJP3* was enriched for the function term, bicellular tight junction. These results indicate that epithelial cells undergo EMT, thereby losing epithelial markers, gaining mesenchymal markers (Vim), and activating fibroblast growth factor receptors (FGFR2, FGFR3, and FGFBP1). Further, some DEGs, i.e., *COL5A1, COL8A1, MMP7*, and *THBS1*, were mapped to extracellular matrix components. To validate the RNA-seq data, we conducted quantitative real-time PCR in Apoe^−/−^ mice (Figure 5C) and found that the expression of *THBS1* in the high-fat group was significantly higher than that in the other top known genes, along with significant overexpression of its receptor CD47 and proteoglycan isoform (GI) of CD47. The high expression of THBS1 and CD47 was confirmed by Western blot and IF (Figures 5D,E). Therefore, we investigated whether *THBS1* is involved in the progression of EMT and inflammation. We silenced *THBS1* expression following transfection of HK-2 cells with the packaged virus (Figure 5F). Although Western blotting revealed that the expression of E-cadherin was not recovered in THBS1 knockdown cells (Figure 5G), the high expression of fibrogenic proteins (α-SMA) and inflammatory genes (TNF-α and IL-1β) significantly decreased in the PLKO-SH group following OX-LDL treatment (Figures 5H,I). These results indicated that THBS1 by itself was not involved in the progression of EMT in OX-LDL-induced injury. To further determine the downstream mechanisms, the CD47-interacting proteins that were immunoprecipitated were subjected to gel electrophoresis and analyzed using LC-MS/MS. We detected 139 proteins in HK-2 cells under OX-LDL stimulation that appeared to interact with CD47; these included γ-catenin and THBS1 (Figure 5J). The IP and IF results supported the co-localization of CD47 and THBS1 and confirmed the colocalization of CD47 with γ-catenin (Figures 5K,L). STRING functional protein association networks (https://string-db.org/) predicted that γ-catenin bound to E-cadherin, which was supported by IP and IF results, and that the expression of E-cadherin decreased following an increase in γ-catenin levels in OX-LDL-treated HK-2 cells (Figures 5M–O). These results suggest that THBS1/CD47 may modulate the interaction between γ-catenin and E-cadherin, and that it may be involved in EMT. This may be the major mechanism by which OX-LDL induces injury. Western blot analysis of renal tissues and HK-2 cells further confirmed this result (Figures 5P,Q). IHC revealed that the expression of CD47, E-cadherin and vimentin increased in high-lipid-challenged mouse models (Figure 5R).

### OX-LDL Promoted EMT and Inflammation via a CD47-Dependent Mechanism in HK-2 Cells

**Figure 6:**
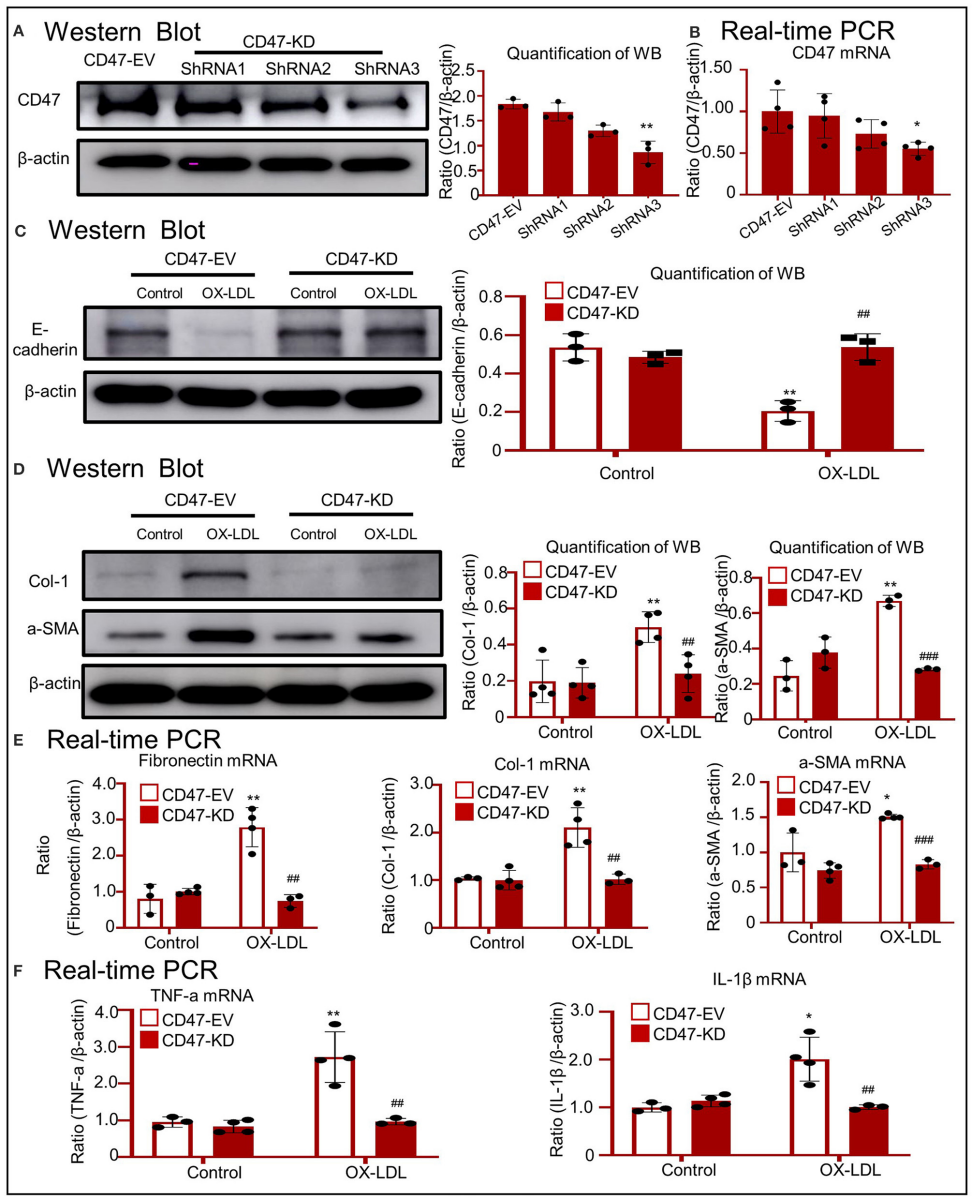
OX-LDL promoted EMT and inflammation via a CD47-dependent mechanism in HK-2 cells. **(A,B)** Verification of CD47 knockdown in HK-2 cells. The results revealed that CD47 expression was downregulated after transfection with CD47 shRNA. **(C)** Western blotting and quantitative data for E-cadherin expression in CD47-silenced HK-2 cells. **(D)** Western blotting and quantitative data for Col-1 and α-SMA expression in CD47-silenced HK-2 cells. **(E)** Real-time PCR for the genes encoding fibronectin, Col-1, and α-SMA in CD47-silenced HK-2 cells. The results demonstrated that CD47 knockdown significantly reduced the mRNA levels of fibrogenic genes. **(F)** Real-time PCR of TNF-α and IL-1β in CD47-silenced HK-2 cells. *p < 0.05, **p < 0.01 as compared with the CD47-EV-control. ##*p* < 0.01, ###*p* < 0.001 compared with the CD47-EV-OX-LDL group. EV, empty vector; KD, knockdown. Data are shown as the mean ± SEM for three to four independent experiments.

To determine the function of CD47 in OX-LDL-treated tubular epithelial cells, we silenced CD47 expression. As shown in Figures 6A,B, the expression of CD47 at mRNA and protein levels was significantly reduced in HK-2 cells. Western blotting showed that the expression of E-cadherin was not significantly affected by OX-LDL in CD47-knockdown HK-2 cells (Figure 6C). Western blotting and real-time PCR revealed that the upregulation of fibrogenic genes was prevented in CD47 plasmid-transfected cells stimulated by OX-LDL (Figures 6D,E). These results were similar to those obtained for the inflammatory markers (Figure 6F), indicating the important role of CD47 in E-cadherin-mediated EMT and inflammation.

### CD47-Targeted Therapy Using Anti-CD47 Antibody Recovered E-Cadherin Expression and Attenuated OX-LDL-Induced EMT and Inflammation in HK-2 Cells

**Figure 7:**
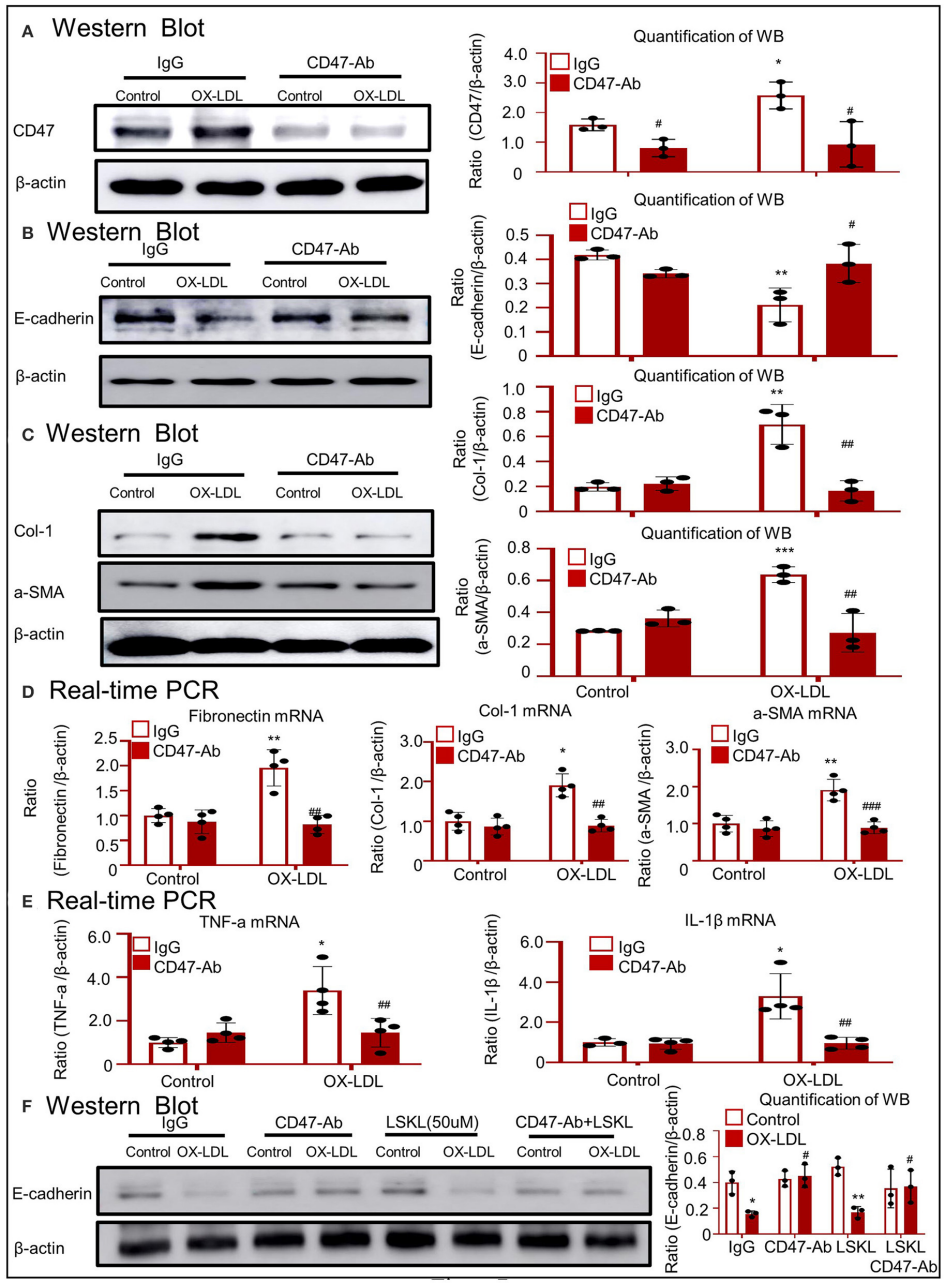
Anti-CD47 antibody attenuated OX-LDL-induced EMT and inflammation in HK-2 cells. **(A)** Western blotting to check CD47 expression. CD47 expression was alleviated in the anti-CD47 antibody treatment group. **(B)** Western blotting and quantitative data for E-cadherin expression. **(C,D)** Western blotting and real-time PCR to check the expression of fibrogenic genes. **(E)** Real-time PCR of inflammatory indices. **(F)** Western blotting to check the expression of E-cadherin. E-cadherin was recovered in the anti-CD47 antibody treatment group and in combination with the LSKL group, but not in the LSKL-independent treatment group. The results indicated that anti-CD47 antibody attenuated OX-LDL-induced EMT and inflammation via a CD47-dependent mechanism in HK-2 cells. *p < 0.05, **p < 0.01, ***p < 0.001 as compared with the IgG control. #*p* < 0.05, ##*p* < 0.01, ###*p* < 0.001 as compared with the IgG-OX-LDL group. Data are shown as mean ± SEM for three to four independent experiments.

Western blotting confirmed that treatment with anti-CD47 antibody failed to upregulate CD47 and disrupt total E-cadherin expression following exposure to OX-LDL (Figures 7A,B). As shown in Figure 7C, the anti-CD47 antibody attenuated OX-LDL-induced expression of Col-1 and α-SMA. These results were supported by the expression of fibronectin, Col-1, and α-SMA mRNA (Figure 7D). Real-time PCR revealed that the anti-CD47 antibody decreased OX-LDL-induced inflammatory marker expression, including TNF-α and IL-1β (Figure 7E). Western blotting revealed that E-cadherin was recovered in the anti-CD47 antibody treatment group and in combination with the LSKL group, but not in the LSKL alone treatment group. The results exclude the role of TGF-β in EMT and indicated that the anti-CD47 antibody attenuated OX-LDL-induced EMT via a CD47-dependent mechanism in HK-2 cells (Figure 7F).

### CD47-Targeted Therapy Using Anti-CD47 Antibody Recovered E-Cadherin Expression and Attenuated EMT and Inflammation in a Type 2 DM Model

**Figure 8:**
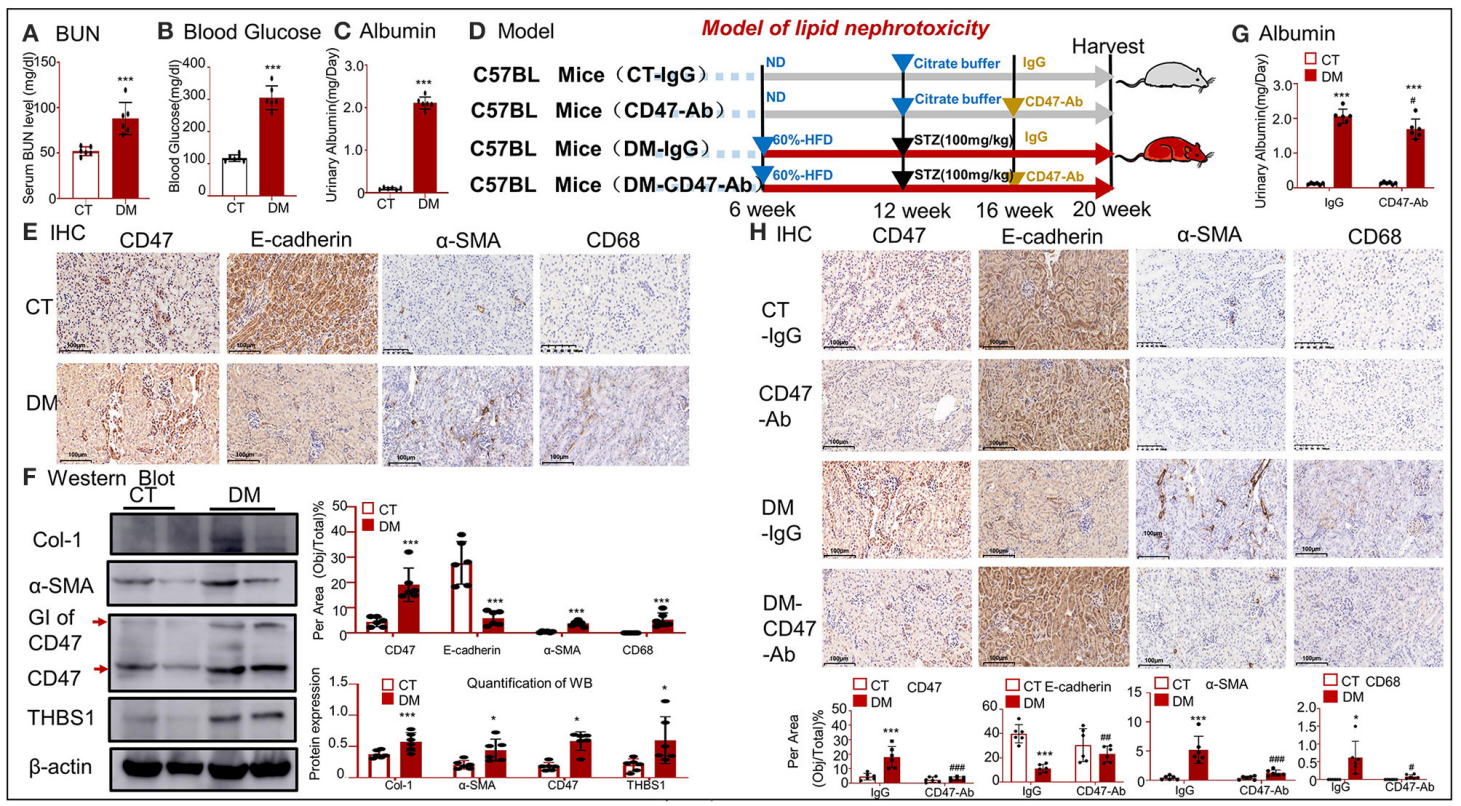
CD47-targeted therapy using anti-CD47 antibody recovered E-cadherin expression and attenuated EMT in type 2 DM. **(A)** Serum BUN; **(B)** blood glucose; **(C)** urinary albumin. **(E)** IHC of α-SMA, CD68, CD47, and E-cadherin. **(F)** Western blotting for Col-1, α-SMA, CD47, and THBS1. Results show that upregulated THBS1/CD47 signaling pathway activated the progression of EMT and infiltration of macrophages in type 2 DM. **(D)** An outline of the protocol used to establish type 2 DM and anti-CD47 antibody treatment. **(G)** Values of urinary albumin. **(H)** IHC of α-SMA, CD68, CD47, and E-cadherin. Results reveal that anti-CD47 antibody mediated blocking of the THBS1/CD47 signaling pathway in type 2 DM decreased the level of urine albumin and attenuated EMT and inflammatory response as compared to type 2 DM. *p < 0.05, **p < 0.01, ***p < 0.001 as compared with CT. #*p* < 0.05, ##*p* < 0.01, ###*p* < 0.001 as compared with the IgG-DM group. CT, control; DM, type 2 diabetes mellitus. Data are shown as mean ± SEM for six to seven independent experiments.

To further confirm the effect of anti-CD47 antibody, another model of hyperlipidemia, i.e., type 2 DM, was established and treated with the anti-CD47 antibody. BUN, blood glucose, and urinary albumin levels significantly increased in the type 2 DM model (Figures 8A–C). H&E staining showed early glomerular lesions in type 2 DM, consisting of focal and segmental areas of mesangial proliferation and expansion (Supplementary Figure 1B). IHC and Western blotting revealed that the upregulated THBS1/CD47 signaling pathway activated the progression of EMT and macrophage infiltration in the type 2 DM model (Figures 8D–F). The treatment of type 2 DM model with anti-CD47 antibody resulted in the blockade of the THBS1/CD47 signaling pathway and consequently attenuated urinary albumin, EMT progression, and inflammatory response (Figures 8G,H). Moreover, the increased inflammatory response observed in type 2 DM was reduced in the DN-CD47 Ab group (Supplementary Figure 1C). These results confirmed that THBS1/CD47 contributed to OX-LDL-mediated EMT.

### CD47-Targeted Therapy Using Anti-CD47 Antibody Attenuated Mitochondrial Oxidative Stress and Apoptosis in a Type 2 DM Model

**Figure 9:**
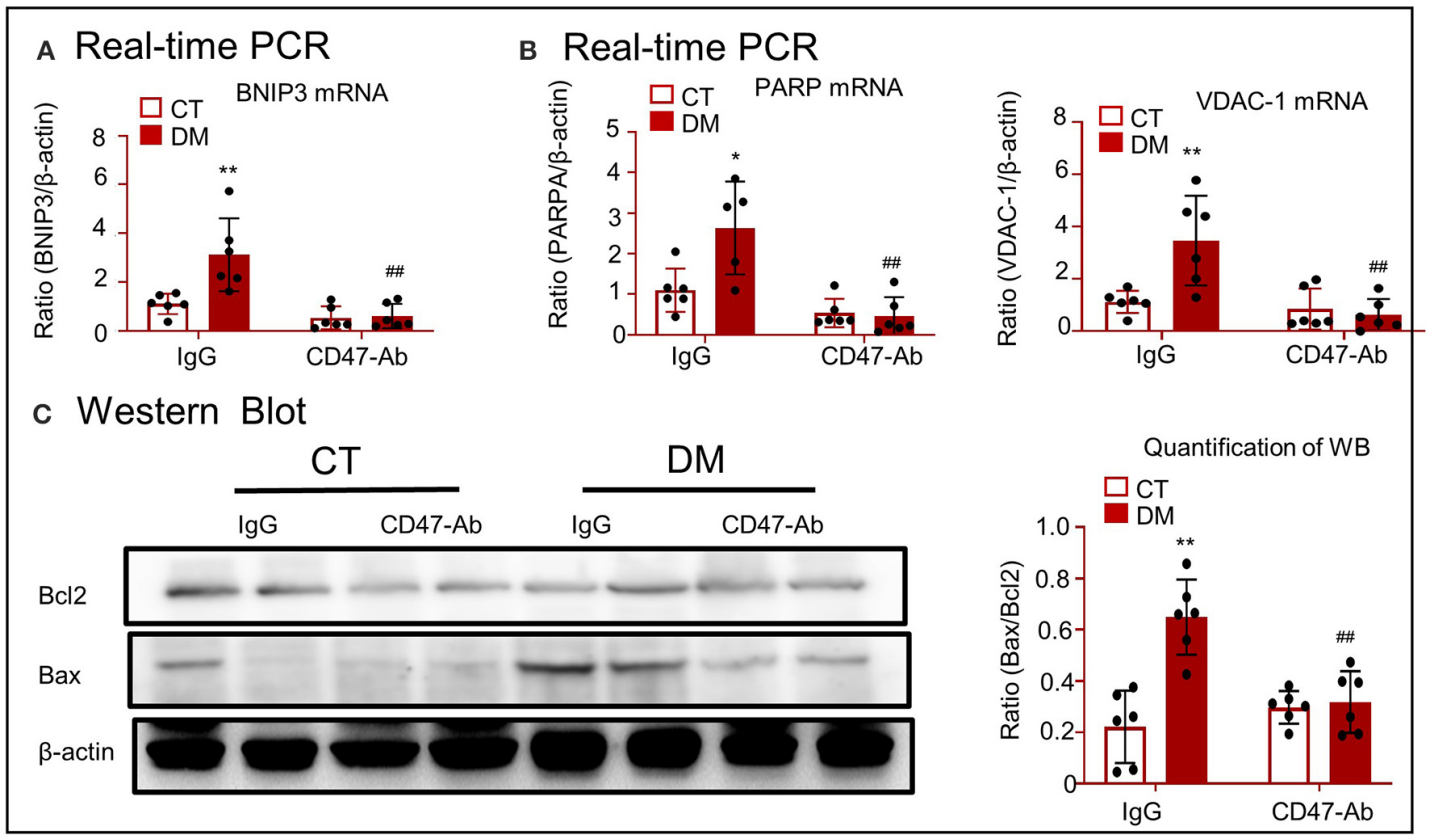
CD47-targeted therapy using anti-CD47 antibody attenuated mitochondrial oxidative stress and apoptosis in type 2 DM model. **(A)** Real-time PCR for checking the expression of *BNIP3*. **(B)** Real-time PCR for checking the expression of *PARP* and *VDAC-1*. **(C)** Western blotting for checking the expression of Bcl2 and Bax. *p < 0.05, **p < 0.01 as compared with CT. ##*p* < 0.01 as compared with the IgG-DM group. CT, control; DM, type 2 diabetes mellitus; PARP, poly (ADP-ribose) polymerase-1; VDAC-1, voltage-dependent anion-selective channel protein 1; Bcl2, B-cell lymphoma 2; Bax, Bcl2 associated X; BNIP3, Bcl2/adenovirus E1B 19-kDa protein-interacting protein 3. Data are shown as mean ± SEM for six to seven independent experiments.

Real-time PCR showed that treatment of type 2 DM model with anti-CD47 antibody resulted in the decrease of BNIP3 mRNA level and consequently attenuated the level of mitochondrial oxidative stress (PARP and VDAC-1), and recovered mitochondrial apoptotic marker (including Bax and Bc12) expression, as compared to the control treatment (Figures 9A–C).

## Discussion

**Figure 10:**
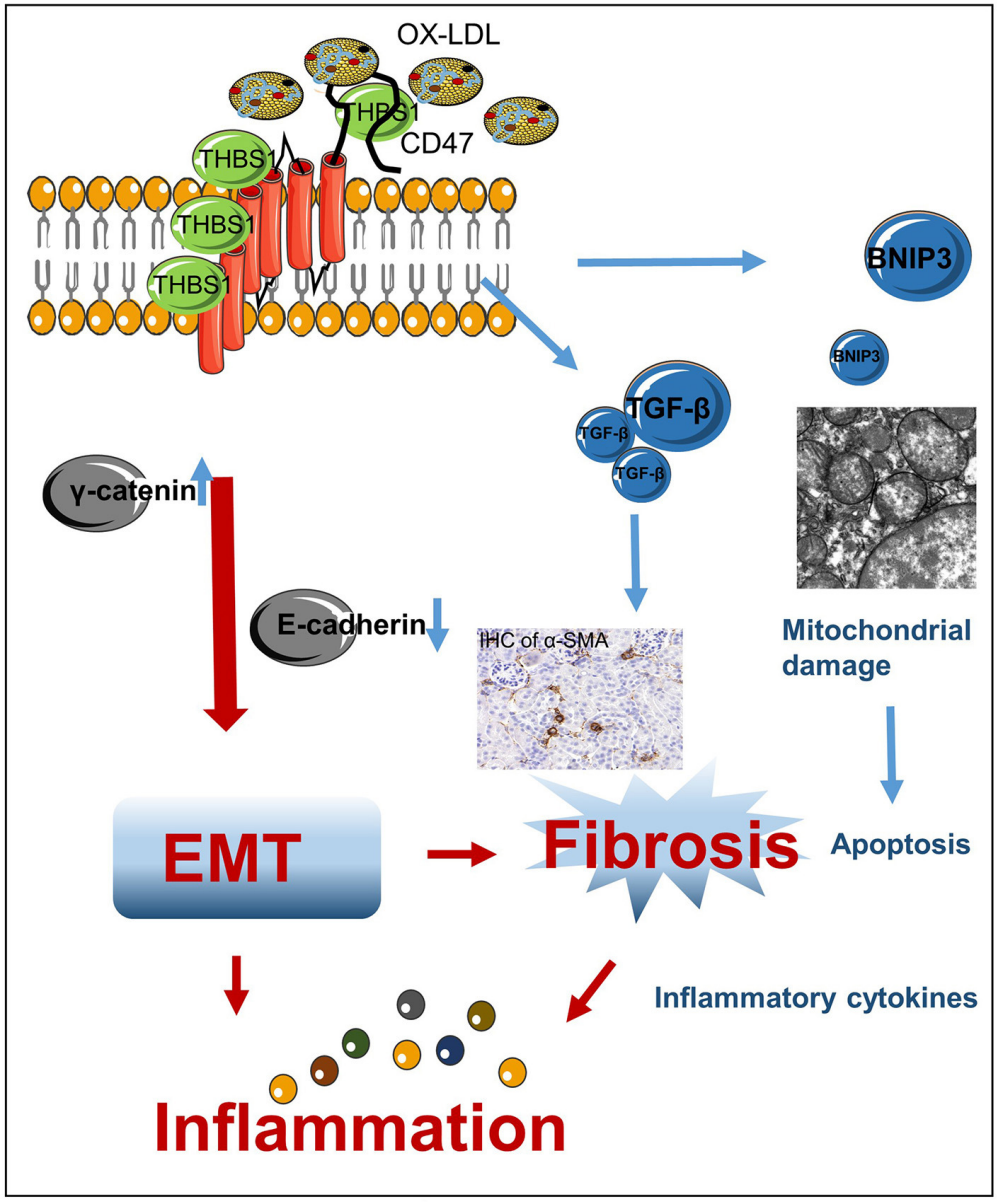
Mechanism of high-lipid-induced kidney EMT and inflammation. High lipid levels induced THBS1/CD47 activation, which modulated the interaction of γ-catenin with E-cadherin and promoted the progression of EMT. This, in turn, resulted in renal fibrosis and inflammation. TGF-β is a secreted cytokine that interacts with THBS1, which may be related to the anti-fibrosis mechanism in OX-LDL-induced kidney injury. BNIP3, a downstream protein of CD47/THBS1, mediates mitochondrial damage and apoptosis.

In the current study, we provide new evidence that THBS1 and CD47 expression increases in the cells or kidney tissues following lipid accumulation. Activation of THBS1/CD47 disrupted the stability of E-cadherin in the plasma membrane of HK-2 cells, thereby accelerating the progression of EMT and triggering an inflammatory response (Figure 10). Silencing of CD47 expression in HK-2 cells or administration of anti-CD47 antibody protected against OX-LDL-induced fibrosis and inflammation *in vitro* and *in vivo* by restoring E-cadherin expression.

Hyperlipidemia is associated with a high incidence of chronic renal disease. Human samples of hyperlipidemia were obtained via rapid autopsy. Experimental evidence showed that high lipid levels induced renal fibrosis in human tissue samples; this result was further confirmed in two HFD-fed mouse models. RNA-seq was conducted to determine the mechanism underlying fibrosis in OX-LDL-induced epithelial injury. The mRNA expression of top known genes was verified in HFD-fed Apoe^−/−^ mice, and the expression of *THBS1* was found to be significantly upregulated in the high-fat group, along with an upregulation in its receptor *CD47*. Among the DEGs mapped to the plasma membrane, several genes were found to be enriched in the biological functions of cell–cell adhesion and bicellular tight junctions. E-cadherin/catenin complexes are important adherence molecules in epithelial tissue. Catenins, including α-catenin, β-catenin, and γ-catenin, are involved in E-cadherin-induced intercellular signal transduction, cell adhesion, and EMT. A recent study showed that CD47 colocalized with E-cadherin at the cell–cell adhesion sites of epithelial cells in HFD-induced kidney disease (Kuwahara et al., 2016). However, the results of LC-MS/MS revealed that CD47 directly interacted with γ-catenin, but not E-cadherin in this study. IP and IF results supported the colocalization of CD47 with γ-catenin (Figures 5K,L). Furthermore, STRING functional protein association networks (https://string-db.org/) predicted that γ-catenin bound to E-cadherin, which was supported by IP and IF, and that the expression of E-cadherin decreased following an increase in γ-catenin in OX-LDL-induced HK-2 cells (Figures 5M–O). These results suggested that THBS1/CD47 modulated the interaction between γ-catenin and E-cadherin and was involved in EMT. It may be the major mechanism by which OX-LDL induces injury. This was further confirmed by CD47 downregulation following transfection with small-hairpin RNA in OX-LDL-treated tubular epithelial cells, and treatment with anti-CD47 antibody in OX-LDL-treated cells and in type 2 DM.

In addition, other genes (*BNIP3, TGF*, and *CD36*) associated with CD47 expression were detected using RNA-seq. Lamy et al. showed that CD47 induced apoptosis through direct interaction with BNIP3 in T cells exposed to phosphatidylserine (Lamy et al., 2003; Ishihara et al., 2013). In this study, although BNIP3 was not included in the results of the LC-MS/MS assay, its expression decreased in type 2 DM mice after anti-CD47 antibody treatment. BNIP3 may be a downstream protein in type 2 DM. BNIP3 has been reported to mediate mitochondrial oxidative stress, apoptosis, and autophagy in acute kidney injury (Ishihara et al., 2013; Tang et al., 2019). Following anti-CD47 antibody treatment, the levels of PARP, VDAC-1, Bax, and Bcl2 recovered, but those of Atg5, Atg7, MDA, and GSH were not restored as compared to those in the untreated control (Figures 9A–C; Supplementary Figures 2D–F). These results show that the anti-CD47 antibody in type 2 DM may decrease BNIP3-mediated mitochondrial oxidative stress and apoptosis. A recent study showed that the transforming growth factor (TGF)-β/mothers against the decapentaplegic homolog 2 (SMAD2) signaling pathway was downregulated in a CD47^−/−^ and anti-CD47 antibody-treated ischemia–reperfusion–nephrectomy (IR-N) mouse model (Julovi et al., 2020). In this study, we performed LC-MS/MS to analyze CD47-interacting proteins and found that TGF-β was included in THBS1/CD47-interacting protein, probably related to the anti-fibrosis mechanism. It was supported by anti-CD47 antibody-mediated blockage of the THBS1/CD47 signaling pathway in type 2 DM (Supplementary Figure 2D). However, E-cadherin was not recovered in the LSKL alone treatment group compared to the anti-CD47 antibody treatment group and in the combination with the LSKL group, which excluded the role of TGF-β in EMT in OX-LDL-induced kidney injury (Figure 7F). OX-LDL exposure results in CD36-mediated cGMP signaling and proliferation in several cell types (Isenberg et al., 2006; Allen et al., 2009; Miller et al., 2010). In 2007, Salajegheh et al. revealed the involvement of the THBS1/CD36/CD47 complex in T cell expansion and inflammatory response to β-amyloid (Salajegheh et al., 2007). In 2010, Miller et al. demonstrated that the interaction of β-amyloid with CD36 results in the induction of CD47-dependent signaling (Miller et al., 2010). A novel study indicated that there is cross-talk between the cell-surface proteins CD36 and CD47–THBS1 in osteoclasts (Koduru et al., 2018). In the present study, although the increase in CD36 expression was detected using RNA-seq, treatment with anti-CD47 antibody failed to decrease the expression of CD36 in type 2 DM. These results suggest that THBS1/CD47 signaling does not contribute to the increase of CD36 in OX-LDL-induced kidney injury. Furthermore, other mechanisms such as cell self-renewal and proliferation (Rogers et al., 2016) and protective innate and adaptive immunity (Navarathna et al., 2015) have been reported to be regulated by CD47. As shown in Supplementary Figure 3, IHC revealed no significant difference in the expression of cMyc, Sox2, and Ki67 between the control and model groups. Hence, self-renewal and proliferation are not the main pathological forms of lipid nephrotoxicity. Treatment with anti-CD47 antibody did not decrease the mRNA levels of *IL4, IL7*, and *IL2* as compared to those in the untreated type 2 DM controls (Supplementary Figure 2A). The mechanism of innate and adaptive immunity also was excluded in OX-LDL-induced kidney injury.

Taken together, we designed two strategies for CD47-targeted therapy to investigate the therapeutic potential of CD47 for lipid nephrotoxicity. ShRNA-mediated CD47 knockdown prevented OX-LDL-induced EMT and inflammation in HK-2 cells. Further, we confirmed the targeted inhibition of CD47 by the anti-CD47 antibody in type 2 DM.

In conclusion, we found that CD47 contributed to lipid nephrotoxicity and that the CD47-targeted therapy protected HK-2 cells from OX-LDL-induced EMT and inflammation. Therefore, CD47 could potentially serve as a novel therapeutic target against lipid-mediated nephrotoxicity.

## Data Availability Statement

The datasets presented in this study can be found in online repositories. The names of the repository/repositories and accession number(s) can be found at: GEO Accession GSE161737.

## Ethics Statement

The studies involving human participants were reviewed and approved by Fourth Affiliated Hospital of Anhui Medical University. The patients/participants provided their written informed consent to participate in this study. The animal study was reviewed and approved by China Pharmaceutical University Ethical Committee.

## Author Contributions

LG performed the experiment, analyzed the data, and wrote the manuscript. T-tY performed the experiment and analyzed the data. J-sZ collected and analyzed tissues from nine patients between 2018 and 2019. C-yH designed, supervised, and reviewed the manuscript. GM designed and supervised the analysis of tissues from nine patients. H-xL, D-cC, L-tW, JW, KG, and X-wL performed the animal experiments. S-yZ, Y-jC, X-xJ, M-mY, and BH provided a series of experimental instructions and help. SW analyzed the LC-MS/MS results. LH, X-yN, and D-mL contributed new reagents and analytic tools. All authors contributed to the article and approved the submitted version.

## Conflict of Interest

The authors declare that the research was conducted in the absence of any commercial or financial relationships that could be construed as a potential conflict of interest.
