# Supplementary figures and images for: THBS1/CD47 Modulates the Interaction of γ-Catenin With E-Cadherin and Participates in Epithelial–Mesenchymal Transformation in Lipid Nephrotoxicity
Source: Front Cell Dev Biol. 2021 Feb 18;8:601521. doi: 10.3389/fcell.2020.601521 (PMC7930485; doi:10.3389/fcell.2020.601521)

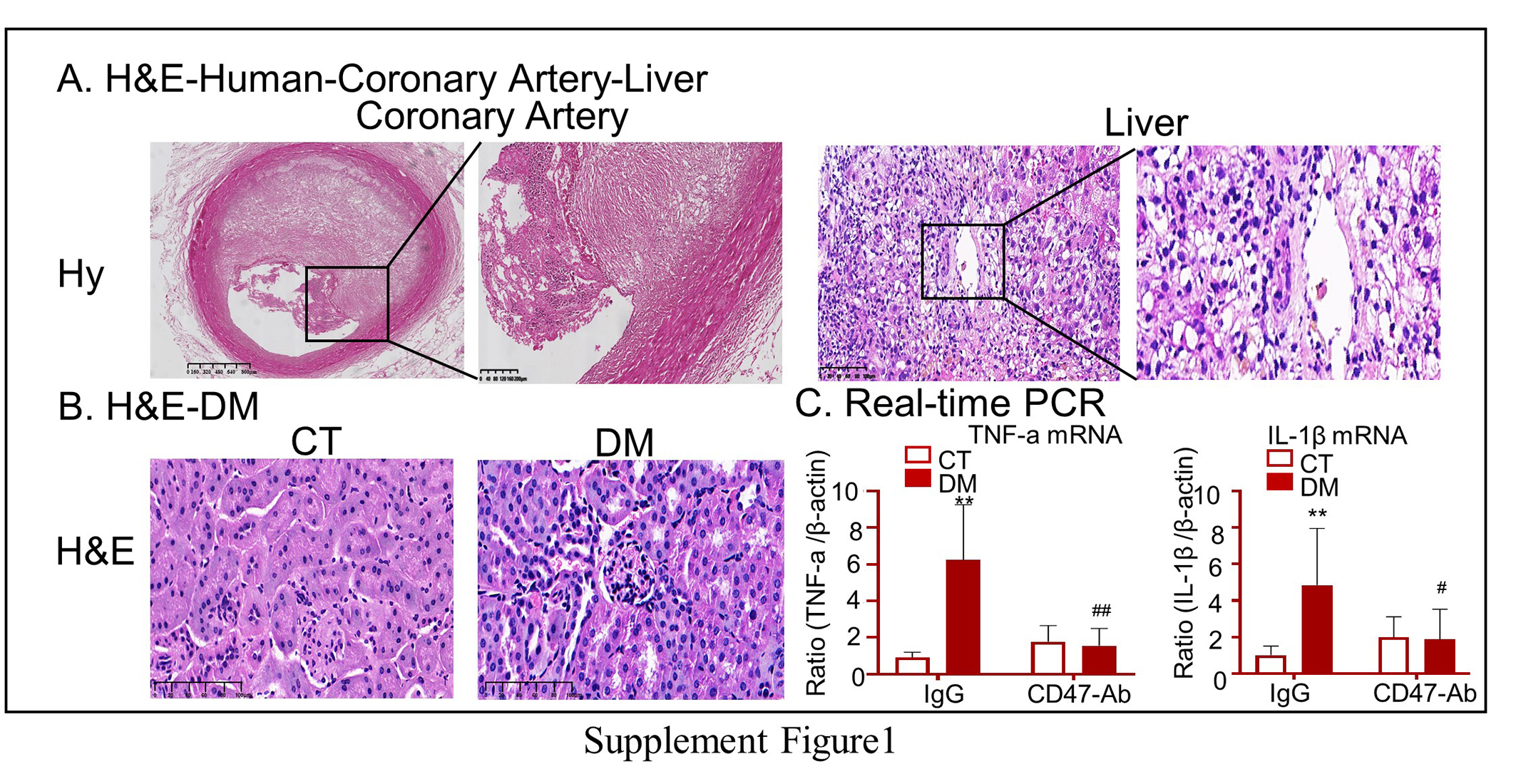

Supplement: Supplementary file 1 [file Image_1.JPEG]

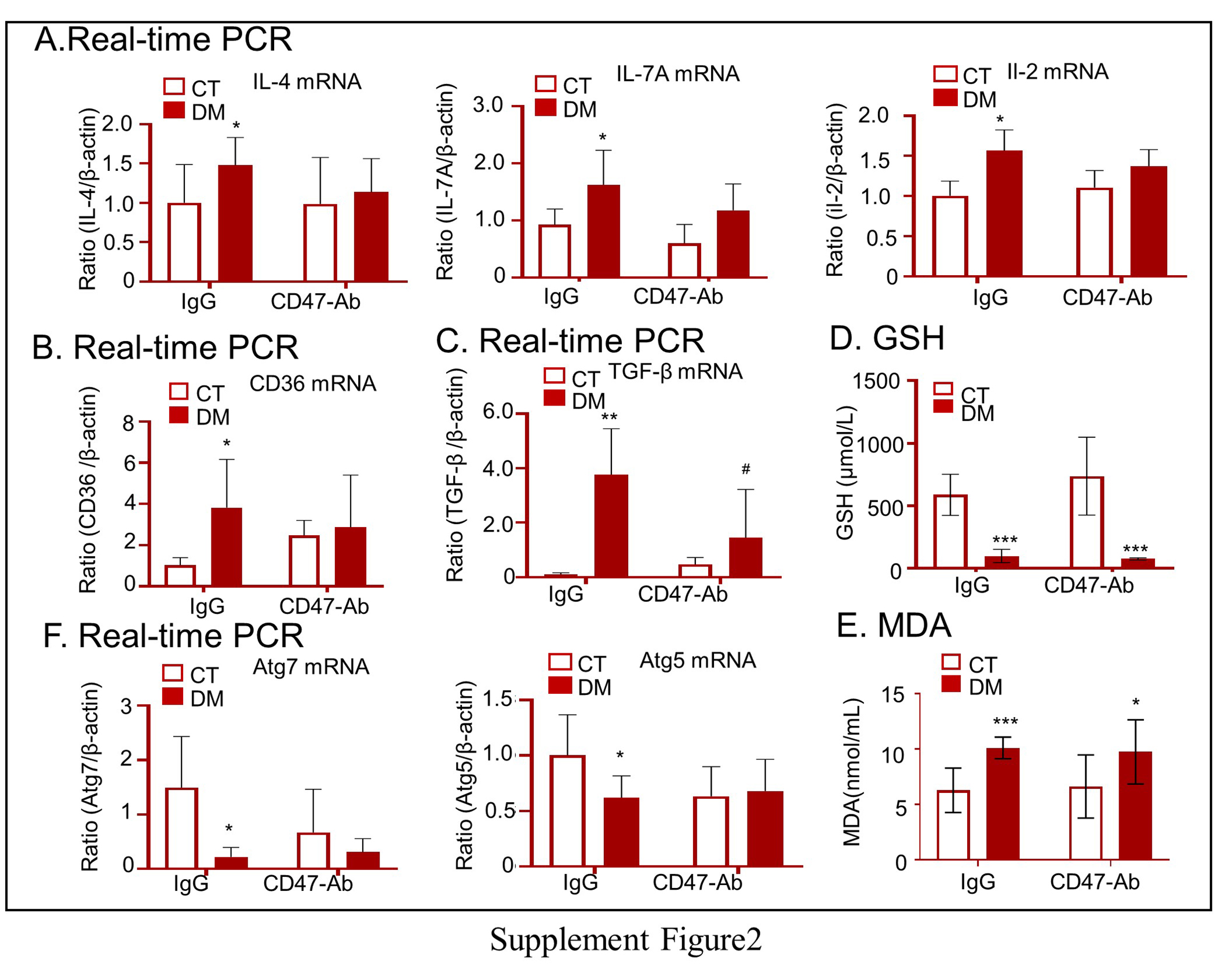

Supplement: Supplementary file 2 [file Image_2.JPEG]

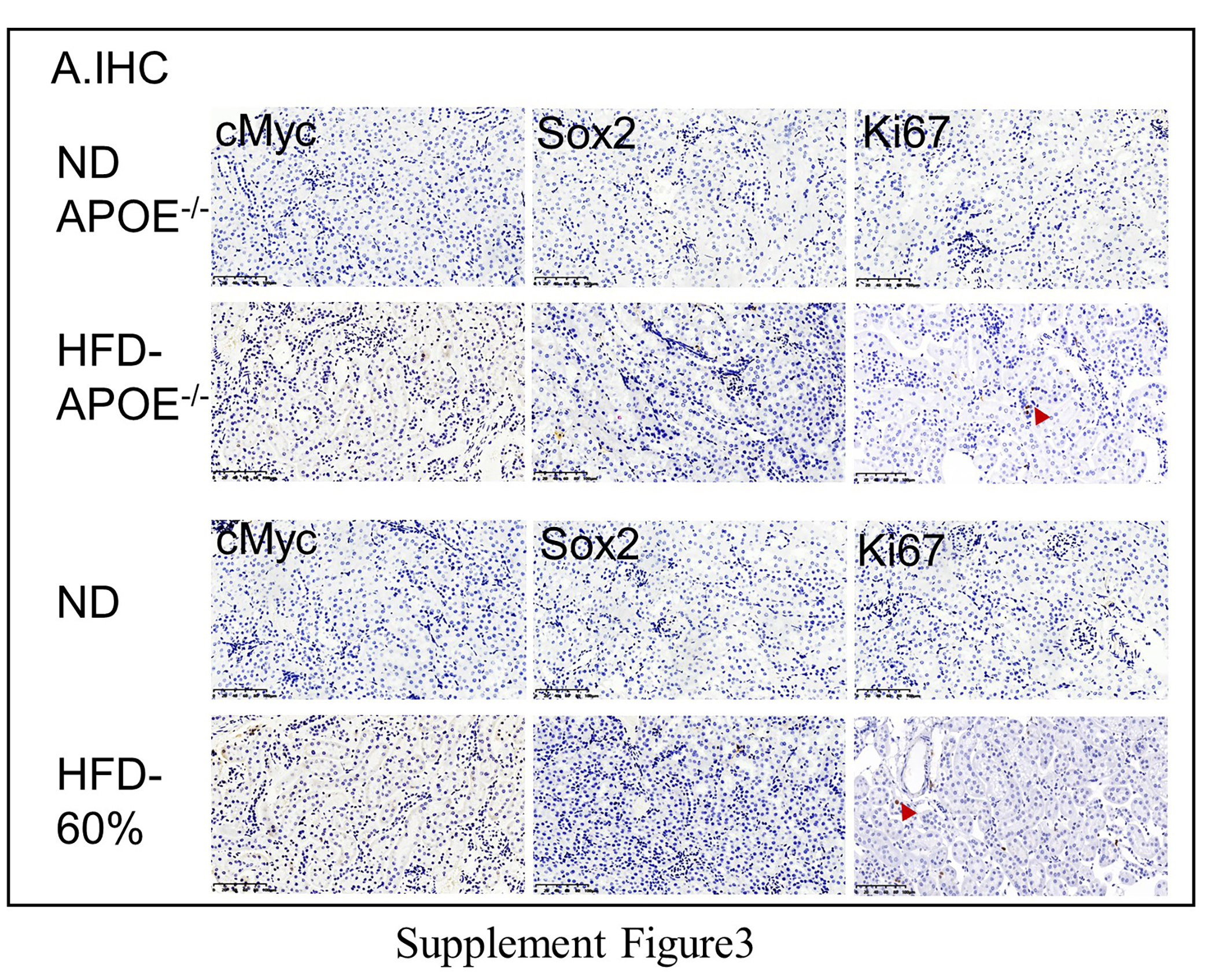

Supplement: Supplementary file 3 [file Image_3.JPEG]
